# Hematologic cancers and infections: how to detect infections in advance and determine the type?

**DOI:** 10.3389/fcimb.2024.1476543

**Published:** 2024-11-04

**Authors:** Yan Chen, Tao Ma

**Affiliations:** Department of Hematology, The Affiliated Hospital of Southwest Medical University, Luzhou, China

**Keywords:** infection, hematologic cancers, procalcitonin, MNGs, biomarkers

## Abstract

Infection is one of the leading causes of death in patients with hematologic cancers. Hematologic cancer patients with compromised immune systems are already susceptible to infections, which come on even more rapidly and are difficult to control after they develop neutrophil deficiencies from high-dose chemotherapy. After patients have developed an infection, the determination of the type of infection becomes a priority for clinicians. In this review, we summarize the biomarkers currently used for the prediction of infections in patients with hematologic cancers; procalcitonin, CD64, cytokines, and CD14 et al. can be used to determine bacterial infections, and (1-3)-β-D-glucan and galactomannan et al. can be used as a determination of fungal infections. We have also focused on the use of metagenomic next-generation sequencing in infections in patients with hematologic cancers, which has excellent clinical value in infection prediction and can detect microorganisms that cannot be detected by conventional testing methods such as blood cultures. Of course, we also focused on infection biomarkers that are not yet used in blood cancer patients but could be used as a future research direction, e.g., human neutrophil lipocalin, serum amyloid A, and heparin-binding protein et al. Finally, clinicians need to combine multiple infection biomarkers, the patient’s clinical condition, local susceptibility to the type of infection, and many other factors to make a determination of the type of infection.

## 1 Introduction

A properly functioning blood system is key to preventing the occurrence of infections in humans, and both neutrophils, monocytes, T-lymphocytes and B-lymphocytes are closely associated with the body’s resistance to infection (Kuraoka et al., 2022; Rousseau et al., 2023). The incidence of most hematologic cancers increases with age (Chen et al., 2022). In 2020, there were 474,519 new cases of leukemia with 311,594 deaths, 544,352 new cases of non-Hodgkin’s lymphoma (NHL) with 259,793 deaths, and 176,404 new cases of multiple myeloma (MM) with 117,077 deaths (Sung et al., 2021). Patients with hematologic cancers have decreased immune function, such as abnormal white blood cell (WBC) function in leukemia, abnormal lymphocyte function in lymphomas, and abnormal plasma cell function in multiple myeloma, and with the fact that patients with hematologic cancers require chemotherapy, chimeric antigen receptor T-cell therapy (CAR-T) or bone marrow transplantation, these patients are susceptible to various types of infections (Bupha-Intr et al., 2021). Infection is one of the main causes of non-cancer deaths among hematologic cancers (Chen et al., 2022). CAR-T therapy results in leukopenia and hypogammaglobulinemia in patients. The overall infection rate in patients with various B-lymphocyte tumors treated with CD19 CAR-T is approximately 40% (Wudhikarn and Perales, 2022). Infection is one of the most common and important causes of death after allogeneic hematopoietic stem cell transplantation(ALLO-HSCT), including bacterial, viral, and fungal infections (Sahin et al., 2016). The main objective of this review is to summarize the various biomarkers of infection in hematologic cancers to help physicians recognize infections in advance as well as to determine the type of infection in order to make timely disposition of patients.

## 2 Biomarkers that have been used in hematologic cancers

### 2.1 White blood cell count

Elevated total WBC counts and neutrophil counts are usually seen in bacterial infections and lacks specificity in stressful situations. However, elevated total leukocyte counts and neutrophil counts in combination with tests such as procalcitonin (PCT) and C-reactive protein (CRP) are also important predictors of bacterial infection in patients (Lavoignet et al., 2019). In leukemia patients, many of whom have abnormally elevated leukocytes at presentation and a marked decline in leukocytes after chemotherapy-induced myelosuppression, even in the presence of bacterial infections, which tend to manifest as febrile neutropenia (FN) during myelosuppression, it is difficult to use leukocytes and neutrophils as a predictor of infection. In children with acute lymphoblastic leukemia presenting with bacteremia, there was no correlation between WBC, CRP, and sedimentation, but doubling of PCT was associated with an odds ratio of 1.32 (95% CI, 1.15-1.53) for positive blood cultures (Vyles et al., 2016). A study by Guo X. Y. et al. (2018) found that neutrophil percentage in leukemia combined with bacterial infection had a sensitivity of 59.25% and a specificity of 85.36%, which was lower than CD64, CRP, and PCT, both in terms of sensitivity and specificity. Hyun S. Y. et al. (2016) studied patients with severe bacterial infections in bortezomib-treated MM and found that poor performance status (Eastern Cooperative Oncology Group ≥ 2), early course of therapy (≤ 2 courses), and pretreatment lymphopenia (absolute lymphocyte count <1.0×10^9^/L) were independent risk factors for severe bacterial infections. Patients with 0, 1, 2, and 3 risk factors had a 5.1%, 14.9%, 23.9%, and 59.5% probability of developing a serious bacterial infection, respectively. Overall, changes in WBC counts have limited application in hematologic cancers.

### 2.2 Procalcitonin

PCT is a precursor of calcitonin, a pro-hormone involved in the inflammatory process. During inflammation, PCT is derived from nearly all cell types, including monocytes and parenchymal tissues, making it a good predictor and diagnostic marker of inflammatory state, with serum levels rising rapidly in inflammation or sepsis (Mangogna et al., 2019). The serum PCT concentration is 0.01-0.05 ng/mL under normal conditions, and the PCT level rises significantly within 2-6 hours after bacterial infection, so it can be used as a predictor of bacterial infection, however, it has some limitations, and it is not specifically up-regulated in major surgery, early post-operative period, trauma, severe burns, inhalation injuries, heatstroke, acute pancreatitis, and cardiac arrest-induced hypothermia and other massive inflammatory states (Choi and McCarthy, 2018; Xu et al., 2022). Yang Y. et al. (2023) evaluated the impact of infection on the prognosis of 1570 patients with malignant hematologic neoplasms and found that a PCT threshold of 0.24 ng/mL predicted in-hospital mortality with a sensitivity of 77.45% and specificity of 59.80%, and advocated for PCT to guide the early recognition and treatment of septic shock. Liu M. et al. (2023) found that leukemia patients were more likely to have positive blood cultures after chemotherapy when their body temperature was greater than 38.95°C and PCT was greater than 0.845 ng/mL. PCT was significantly increased in leukemia patients with blood infections. On the day that microbial growth was first detected in blood cultures, PCT levels were significantly higher in patients with gram-negative bacteremia (G-) (4.110 ng/mL) than in patients with gram-positive bacteremia (G+) (1.020 ng/mL), and all patients had G- when the PCT concentration was >10.000 ng/ml (Gu et al., 2020). There were no differences in the immediate outcome measures of CRP and PCT between patients with and without antimicrobial prophylaxis after chemotherapy in leukemia patients, but the median duration of fever, CRP, and PCT values were higher in patients with antimicrobial prophylaxis (Chan et al., 2021). The benefit of antimicrobial prophylaxis after chemotherapy has received less support. There is a weak positive correlation between PCT and Sequential Organ Failure Assessment (SOFA) scores, and PCT also differentiated between G- and G+ infections, with PCT levels being higher in G+ than in G- on the third day of fever, which may be associated with a more rapid response to treatment in G- patients (Moustafa et al., 2021). CRP and PCT are associated with multiple organ dysfunction syndrome (MODS) and death, respectively, in pediatric children with hematological disorders and tumors receiving chemotherapy (Lin et al., 2024). Both PCT and CRP are useful biomarkers for predicting bacteremia, but PCT may be a better early biomarker for predicting bacterial infection at the time of onset of FN in children with acute leukemia (AL) than CRP (Nahar et al., 2023).

Hodgkin’s lymphoma (HL) is diagnosed with elevated inflammatory mediators and 85% of patients present with elevated CRP, but the inflammatory properties of HL are not associated with elevated PCT, and a normal PCT rules out bacterial infection (Piperidou et al., 2022). In 212 cases of NHL, patients with microbiologically documented infections (MDIs) had higher initial PCT values compared with patients with fever of unknown origin, patients with initial PCT values ≥ 0.50 ng/mL were at high risk for MDIs, and elevated PCT was strongly associated with patient mortality (Liu et al., 2015). Bloodstream infections caused by Gram-positive bacterial multidrug-resistant (MDR) strains have significantly higher PCT levels than non-MDR strains (Luo et al., 2019). Early febrile events in 62 patients with relapsed/refractory B-cell NHL treated with CD19-CAR-T were characterized as infections or cytokine release syndrome (CRS), and PCT was particularly high in infected patients compared with CRS (optimal threshold for discrimination was 1.5 µg/L) (Rejeski et al., 2023). Autologous hematopoietic stem cell transplantation (ASCT) for MM may be associated with infection and engraftment syndrome (ES), both of which are associated with fever and elevated CRP, but only patients with bacteremia had elevated PCT levels, whereas all other patients had PCT levels below 2 ng/mL, with a PCT threshold of <2 ng/mL, which can be used to identify patients with ES-associated noninfectious fever after ASCT (Knoll et al., 2019).

Undoubtedly, PCT is a good predictor of bacterial infections in hematologic patients, but it is still important to be aware of nonspecific elevated PCT, which need to be judged in the context of the patient’s history, symptoms, and signs.

### 2.3 Neutrophil CD64

CD64 is a high-affinity immunoglobulin (Ig)-G Fc receptor (Fcγ RI) characterized by rapid and strongly induced expression on neutrophils in response to infection or the pro-inflammatory cytokines interferon (IFN)-γ and granulocyte colony-stimulating factor (G-CSF), and it is an early biomarker for the diagnosis of sepsis in patients, especially bacterial infection (Cid et al., 2010; Liu Q. et al., 2022). CD64 is mainly distributed in monocytes, macrophages, myeloid precursors and dendritic cells, while it is not expressed in lymphocytes, erythrocytes and megakaryocytes. Detection of CD64 requires the use of a flow cytometer, and Liu Q’s article describes in detail the steps for CD64 detection (Liu Q. et al., 2022). Neutrophil (n) CD64 is significantly higher in both bacterial and viral infections than in fungal infections, but does not distinguish between bacterial and viral infections. In bloodstream infections, the nCD64 index is significantly higher in G-type bacterial infections than in G+-type bacterial infections (Liu Q. et al., 2022). However, patients with lymphoma may themselves have elevated expression of nCD64, which needs to be differentiated from infections (Komiya et al., 2016; Liu Q. et al., 2022).

In AL, nCD64 has a sensitivity of 88% and a specificity of 80% as an indicator of infection and can be used as a diagnostic indicator in patients with leukemia combined with early infection (Guo et al., 2016). In another study, nCD64 was found to have a sensitivity of 71.06% and a specificity of 91.46%, which was superior to CRP, PCT, and neutrophil percentage, as an indicator of infection in patients with leukemia combined with bacterial infection. It helps in the early diagnosis of the disease (Guo et al., 2018). nCD64 is an inflammatory cytokine expressed by neutrophils after exposure to a large number of inflammatory cytokines (e.g., lipopolysaccharide, G-CSF, etc.), and although it has been shown in the literature to have a predictive role in infections in patients with leukemia, the changes of nCD64 in granulocyte-deficient patients still need to be further investigated. nCD64 may have some application in the diagnosis of early infection in leukemia, but cannot be used as an indicator of infection in lymphoma.

### 2.4 Cytokines

Activated immune cells produce cytokines that enhance host defenses against bacterial and viral infections, and in recent years cytokines have played an important role in the diagnosis of infections (Akdis et al., 2016). In pediatric hematology patients, IL-6 and IL-10 have a good predictive value for the diagnosis of serious infections (Lin et al., 2024). In pediatric patients with hematologic cancers, Xia t. et al. (2016) analyzed 3023 samples (2819 fever case samples and 204 control samples) and found that the positivity rates of interleukin-6 (IL-6) and IL-10 in children with bacteremia were 92.8% and 82.2%, respectively, which were higher than those of PCT (33.8%) and CRP (73.1%). The specificity of IL-6 and IL-10 was significantly higher than that of PCT in the diagnosis of G- bacteremia. Zhang L et al. (Zhang L. et al., 2022) performed cytokine assays in 229 newly diagnosed NHL patients and 40 healthy adults, and found that IL-6, IL-8, IL-10, tumor necrosis factor (TNF)-β, IFN-γ, CRP, and PCT levels were higher in NHL patients with respiratory infections than in those without infections, and patients with both respiratory infections and bacteremia had higher IL-6. IL-6 ≥ 18.79 pg/mL indicated the presence of a pulmonary bacterial infection in patients, whereas IL-6 ≥102.6 pg/mL may suggest pulmonary bacterial infection with bacteremia.

IL-6 also recognizes whether fever in CART therapy is caused by infection or CRS. In a study of CD-19 CART for relapsed refractory B-cell lymphoma, patients who developed severe infections had elevated IL-6 levels on the day of the event compared with the CRS-only cohort (median 2243 pg/mL versus 64 pg/mL, P = 0.03) (Rejeski et al., 2023). In patient who developed infections during the myelosuppressive phase after chemotherapy for lymphoma, IL-6 and IL-10 expression was significantly elevated in patients with G- bacterial infections, whereas IL-6 expression was elevated in patients with G+ infections, which helped to differentiate between G- and G+ bacterial infections at an early stage (Zhu et al., 2022). Patients with acute lymphoblastic leukemia (ALL) have an inherently intense, dysregulated inflammatory state characterized by Th1 polarization, with higher levels of TNF-α, IL-6, IL-8, monocyte chemotactic protein-1 (MCP-1), and IL-10 in patients with ALL compared to controls (Pérez-Figueroa et al., 2016). However, cytokines still play an important role in predicting patients with infections. IL-6, IL-10 and TNF-α levels were significantly higher in patients with leukemia combined with bacterial infection than in patients with AL without combined infection, and serum IL-6, IL-10 and TNF-α levels were significantly higher in the septic shock group than in the patients in the mild-to-moderate infection group (Zhang WF. et al., 2022). In chronic myeloid leukemia (CML), TKI treatment did not affect cytokine expression in patients, and 83.33% of CML patients with pulmonary bacterial infections had IL-6 ≥ 13.78 pg/ml, whereas the probability of pulmonary bacterial infections was 93.55% when levels of IL-6, IL-8, and IL-10 exceeded the critical threshold at the same time (Guan et al., 2023). In MM patients treated with bortezomib, there is an imbalance in the Th1/2 ratio, making them more susceptible to bacterial and viral infections (Li et al., 2015). In another study, a lower Th1/Th2 ratio and higher cytokine response ratios of IL-5 and IL-13 were found to be significantly associated with an increased risk of infections during maintenance therapy in MM patients (Teh et al., 2017). Cytokines are a response to the inflammatory state of the body. Cytokines tend to be markedly elevated, particularly IL-6 and IL-10, following co-infections in hematologic neoplasms, which has implications for predicting early infections and differentiating between G- from G+.

### 2.5 Presepsin

CD14 is a coreceptor that is consistently expressed on the surface of monocytes/macrophages. There are two forms of CD14: membrane-bound CD14 (mCD14) and soluble CD14 (sCD14). Presepsin (PSPN) is derived from sCD14, and plasma levels of PSPN can be considered an indicator of activation of innate immune effector cells in response to invading pathogens (Memar and Baghi, 2019). PSPN secretion stimulates phagocytosis by monocytes (Chenevier-Gobeaux et al., 2015). Sixty AL patients developed fever after chemotherapy, and the critical values of PSPN were 1.75 and 2.9 μg/L during the first and third days of fever, respectively, and it had a high clinical sensitivity. The difference in PSPN values between the different patient groups (fever of unknown origin, localized infection and bacteremia) was statistically significant (p<0.05), and the difference in PSPN between infected and uninfected patients was statistically significant (3.7 μg/L and 2.4 μg/L, respectively, p = 0.001) (Moustafa et al., 2021). However, there was no significant correlation between PSPN values and SOFA scores (Moustafa et al., 2021). In pediatric hematological neoplasms, the increment of PSPN was higher in blood culture-positive cases than in sterile cases. PSPN concentrations were significantly higher in patients with bacteremia than in patients with clinically confirmed infection and unexplained fever (Baraka and Zakaria, 2018). PSPN is a useful indicator for the detection of bacteremia with a sensitivity and specificity of 100% and 85.7%, respectively (Baraka and Zakaria, 2018). Patients presenting with fever after hematopoietic stem cell transplantation (HSCT) had the highest diagnostic value for PSPN compared with PCT and CRP, with a threshold value of 218 pg/mL for adult patients with suspected G- stream infection after HSCT (Stoma et al., 2017). PSPN was more effective in the diagnosis of hemophagocytic syndromes (HPS) occurring in hematological malignancies; its optimal threshold as well as sensitivity and specificity were 722 pg/mL, 92% and 97%, respectively (Koh et al., 2017). PSPN, although present on the surface of monocytes, showed good predictability of infection both in AL and in HSCT, even though these patients were neutropenic. PSPN can be used as a biomarker of bacterial infection in patients with hematologic cancers and can also indicate the presence of HPS.

### 2.6 Caspase-cleaved cytokeratin-18

Caspase-cleaved cytokeratin (CCCK)-18 is a protein released into the bloodstream during apoptosis. At the time of sepsis diagnosis, circulating concentrations of CCCK-18 were higher in septic patients who die than in those who survive, and there was an association between sepsis severity and mortality and serum CCCK-18 levels in the first week (Lorente et al., 2017). Intke C et al. (Intke et al., 2022) investigated the role of CCCK-18 in the development of FN in patients with hematologic disorders. A total of 86 patients were enrolled, of whom 23 had acute myeloid leukemia (AML), 43 had NHL, 17 had MM, 3 had Hodgkin’s lymphoma (HL), and 63 underwent ASCT. The study found that higher CCCK-18 levels were associated with severe sepsis, intensive care unit treatment, and mortality outcomes, but not with positive blood cultures. The optimal threshold for CCCK-18 fragment M30 to predict severe sepsis was 205 U/L on day 1 of fever.

### 2.7 (1-3)-beta-D-glucan

(1-3)-β-D-glucan (BDG), a component of fungal cell walls, has been shown to stimulate the immune system, enhance hematopoiesis, amplify killing of eosinophilic tumors, increase neutrophil chemotaxis and adhesion, and may be used in the diagnosis of invasive fungal infections (IFI) (Weitberg, 2008; Liss et al., 2016).

BDG plays an important role in predicting fungal infections in patients with hematologic cancers. Possible causes of BDG false positives include hemodialysis membranes, blood products, use of antineoplastic and antimicrobial drugs and IgG-type MM (Mori et al., 2010; Issa et al., 2012; Liss et al., 2016). Issa NC et al. found that BDG in patients with MM or Waldenstrom’s macroglobulinemia (WM) does not result in false positives and can be used as a biomarker in patients with IFI, but patients with plasma cell disorders and IgG levels greater than 2,000 mg/dl have a higher chance of having an undecipherable BDG result (Issa et al., 2012).

Of 190 consecutive neutropenic AL cases in 95 patients, the BDG test diagnosed 30 confirmed or probable IFIs, with sensitivities, specificities, positive predictive values, negative predictive values, and efficiencies of two consecutive BDG values ≥7 pg/mL for confirmed or probable IFIs of 0.63, 0.96, 0.79, 0.91, and 0.89, respectively (Senn et al., 2008). BDG is available for both the Dynamiker^®^ Fungal (1-3)-β-D-Glucan Assay (DFA) and the well-established Fungitell^®^ Assay (FA), with the DFA and FA performing highly consistently in quantitative and qualitative terms, with 85% concordance between the two, and ≥2 consecutive positive samples are used as a diagnostic in a high-risk setting for patients with hematological malignancy criteria for IFI, a higher negative predictive value was found (Siopi et al., 2022). As an adjunctive diagnostic method for IFI, BDG has high sensitivity and specificity in patients with AML and myelodysplastic syndrome (MDS), absence of a positive BDG finding had a 100% negative predictive value, and the specificity of the test was 90% for a single positive test result and >or=96% for >or=2 sequential positive results (Odabasi et al., 2004).

Elevated BDG is a good predictor of IFI in patients with hematologic cancers, but attention needs to be paid to its false-positive profile and the effect of elevated IgG on it.

### 2.8 Galactomannans

Serum galactomannan (GM) testing is primarily used for the diagnosis of invasive aspergillosis (IA) (Tong et al., 2018). The false-positive rate of serum GM testing is higher in older patients with higher serum IgG levels (Abe et al., 2019). It has also been suggested that MM correlates with the rate of false-positive GM tests, independent of monoclonal protein levels or immunoglobulin type (Ko et al., 2016). In patients with hematologic malignancies, among patients with probable/possible/no IA, the positivity rates were 88%/8%/0% (*p* < 0.001) for GM, 62%/46%/35% (p = 0.29) for BDG, and 62%/33%/27% (p=0.15) for *Aspergillus* DNA (PCR) (Siopi et al., 2021). Most GM+ and PCR+ samples appeared in the first and second week of clinical evaluation, while BDG appeared later, and the investigators advocated the use of GM in combination with PCR for the early diagnosis of IA (Siopi et al., 2021). Risk factors for early death after Invasive mold infections (IMI) in patients with hematologic cancers include elevated serum GM, treatment with amphotericin B, deterioration at 3 weeks after diagnosis of IMI, and HSCT for lymphoma, with late death associated with recurrent/refractory malignancy and elevated serum GM (Yashphe et al., 2021). In children with ALL, GM can be detected in serum before clinical signs of IA appear, but its sensitivity and specificity vary (Avcu et al., 2017). False positivity of GM is a distinct disadvantage, and consecutively positive GM must be considered in the presence of clinical and imaging findings associated with IA (Avcu et al., 2017). Serum GM test is mainly used as a biomarker for predicting IA, and we also need to pay attention to the problem of its false-positive rate, which should be determined comprehensively in combination with imaging and clinical related data.

The above sections are routine biomarkers of infection that have been used in blood cancers and we summarize them in **Table 1**.

**Table 1:** Biomarkers for predicting infections.

Biomarkers	Changes in biomarkers	Hematologic cancers	References
WBC	WBC and neutrophil counts are usually elevated when bacterial infection occurs	leukemia	(Guo et al., 2018)
Lymphopenia	absolute lymphocyte count <1.0×10^9^/L	bortezomib-treated MM	(Hyun et al., 2016)
PCT	PCT threshold of 0.24 ng/mL predicted in-hospital mortality with a sensitivity of 77.45% and specificity of 59.80%	AL, MM, Diffuse large B-cell lymphoma	(Yang et al., 2023)
PCT	The threshold values of 38.95°C for febrile body temperature and 0.845 ng/mL for PCT suggest a greater likelihood of positive blood cultures	leukemia	(Liu M. et al., 2023)
PCT	PCT was higher in patients with G- than in G+. A PCT threshold of 10.000 ng/mL was found in all cases of G-	leukemia	(Gu et al., 2020)
PCT	A normal PCT can rule out bacterial infection	HL	(Piperidou et al., 2022)
PCT	PCT was particularly high in patients who developed infections compared to CRS (optimal threshold for discrimination was 1.5 µg/L)	relapsed/refractory B-cell NHL treated with CD19-CAR-T	(Rejeski et al., 2023)
PCT	PCT threshold less than 2 ng/mL can be used to identify patients with ES-associated noninfectious fever after ASCT	MM who received ASCT	(Knoll et al., 2019)
nCD64	nCD64 has a sensitivity of 88% and a specificity of 80% as an indicator of infection	AL	(Guo et al., 2016)
nCD64	nCD64 has a sensitivity of 71.06% and a specificity of 91.46% as an indicator of infection	AL	(Guo et al., 2018)
IL-6 and IL-10	In bacteremia, IL-6 and IL-10 were more positive than PCT and CRP, and IL-6 and IL-10 were significantly more specific than PCT in the diagnosis of diagnostic G- bacteremia.	Pediatric patients with hematologic cancers	(Xia et al., 2016)
IL-6	IL-6 ≥ 18.79 pg/mL indicated the presence of pulmonary bacterial infection in patients, and IL-6 ≥102.6 pg/mL may suggest pulmonary bacterial infection with bacteremia.	NHL	(Zhang L. et al., 2022)
IL-6 and IL-10	The expression of IL-6 and IL-10 was significantly elevated in patients with G- bacterial infections, and IL-6 was elevated in patients with G+ infections	Lymphoma	(Zhu et al., 2022)
IL-6, IL-10 and TNF-α	IL-6, IL-10 and TNF-α levels were significantly higher in patients with leukemia combined with bacterial infection than in patients with AL without combined infection	AL	(Zhang WF. et al., 2022)
IL-6	83.33% of CML patients with pulmonary bacterial infections had IL-6 ≥ 13.78 pg/ml	CML	(Guan et al., 2023)
IL-6	IL-6 levels were elevated on event day in patients who developed severe infections compared with the CRS-only cohort (median 2243 pg/mL versus 64 pg/mL, P = 0.03)	Relapsed/refractory B-cell NHL treated with CD19-CAR-T	(Rejeski et al., 2023)
Th1/2 ratio	An imbalance in the Th1/2 ratio leads to a greater susceptibility to bacterial and viral infections.	MM patients treated with bortezomib	(Li et al., 2015)
Th1/Th2 ratios, IL-5 and IL-13	Lower Th1/Th2 ratios and higher cytokine response ratios for IL-5 and IL-13 were significantly associated with an increased risk of infection	MM patients on maintenance therapy	(Teh et al., 2017)
Presepsin	The difference in PSPN between infected and uninfected patients was statistically significant (3.7 μg/L and 2.4 μg/L, respectively, p = 0.001)	AL	(Moustafa et al., 2021)
Presepsin	PSPN is a useful indicator for the detection of bacteremia with a sensitivity and specificity of 100% and 85.7%, respectively	Pediatric hematological neoplasms	(Baraka and Zakaria, 2018)
Presepsin	Threshold of 218 pg/mL in adult patients with suspected G- stream infection after HSCT	Patients who underwent HSCT	(Stoma et al., 2017)
Presepsin	Its optimal threshold as well as sensitivity and specificity were 722 pg/mL, 92% and 97%, respectively	HPS occurring in hematological malignancies	(Koh et al., 2017)
Caspase-cleaved cytokeratin-18	The optimal threshold for CCCK-18 fragment M30 to predict severe sepsis was 205 U/L on day 1 of fever	Patients with hematologic neoplasms who develop FN	(Intke et al., 2022)

## 3 Metagenomic next-generation sequencing

Next-generation sequencing (NGS) is a genre of technology that allows independent sequencing of thousands to billions of DNA fragments simultaneously. Almost all infectious pathogens contain DNA or RNA genomes, which makes sequencing an attractive method for pathogen detection, and the applications of NGS in clinical microbiology are multifaceted, including metagenomic NGS (mNGS), which allows for the detection of pathogens in an unbiased manner (Gu et al., 2019). There are many reports in the literature on the successful use of mNGS to detect pathogens after secondary infection in patients with AL (Wen et al., 2021; Chang et al., 2023). Qi Y et al. retrospectively studied 37 patients with FN and compared the results of plasma mNGS with blood culture (BC). They found that the diagnostic positivity rate of mNGS (25/37, 67.6%) was significantly higher than that of BC (7/37, 18.9%), and that the early implementation of mNGS could effectively improve the efficiency of pathogen detection in patients with AL FN (Qi et al., 2023). Zhang P et al. recruited 147 children with hematologic malignancies in combination with fever who had no clear microbial etiology on routine testing. Of these, 112 (76.2%) had at least one microorganism identified by mNGS, 48 patients had their initial antimicrobial regimen adjusted based on the mNGS results, and of these, 41 had remission of their febrile symptoms, with a total of 27.9% of patients (41/147) benefiting from mNGS (Zhang P. et al., 2022). Feng S et al. recruited 442 adult patients with AL combined with FN and found that the positive and negative agreements of mNGS compared to BC were 81.91% (77 of 94) and 60.92% (212 of 348), respectively. 81 (36%) of 225 mNGS-positive cases received antimicrobial adjustments, with a positive impact on 79 patients (Feng et al., 2024). Further analysis showed that mNGS was less affected by prior antibiotic exposure than BC (Feng et al., 2024). The mNGS analysis shows good diagnostic potential in rapidly identifying clinically relevant pathogens, but may also detect many clinically irrelevant pathogens, patients with higher IL-6 (≥ 390 pg/ml) are likely to have bacterial infections, and the integration of IL-6 improves the accuracy of interpretation of mNGS results (Wang et al., 2022). The mNGS also has excellent clinical applications in blood cancer patients with fungal infections. Diagnosis of invasive pulmonary aspergillosis (IPA) by peripheral blood mNGS is also well used in clinical work. Ma X et al. found that 5 patients (2 MDS, 2 AML, 1 renal transplantation) were positive for *Aspergillus* DNA by peripheral blood mNGS, and they were subsequently diagnosed with IPA by other methods such as lung histopathology, bronchoalveolar lavage fluid (BALF) mNGS, sputum culture or sputum mNGS (Ma et al., 2022). Detection of *Aspergillus* DNA by peripheral blood mNGS can be used to diagnose IPA and is a rapid, noninvasive diagnostic method (Ma et al., 2022). For infections of the central nervous system, mNGS can be performed on cerebrospinal fluid or drainage fluid from brain abscesses to clarify the type of infection (Wei et al., 2024). What is the diagnostic accuracy of mNGS in plasma and blood cell samples in patients with FN? It was found that plasma and blood cell mNGS identified the causative pathogens in 58 and 46 cases, respectively, with positivity rates of 53.7% and 76.7% (Wang et al., 2024). Plasma mNGS had a high sensitivity but a high rate of false positives, while blood cell mNGS had a low sensitivity but a high specificity (Wang et al., 2024). Jin D et al. performed conventional laboratory microbiological testing of 15 lymphoma patients with chemotherapy-associated interstitial pneumonitis (IP) and mNGS for BALF, and found that the microbial detection rate of mNGS was 93.3% (14/15) compared to 13.3% (2/15) with conventional culture methods, and that the application of mNGS had a higher sensitivity in patients with chemotherapy-associated IP (Jin et al., 2023). The mNGS assay has excellent clinical value in patients with hematologic cancer co-infections, but sometimes mNGS detects multiple microorganisms, and the combination of mNGS with cytokines and other biomarkers of infection to guide antibiotic selection is a good direction for research.

## 4 Future biomarkers for hematologic cancers

Many biomarkers have been used as predictors of infection in humans. Hematologists also use these indicators in the clinic to help identify the type of infection and guide treatment. These biomarkers are the future direction of research in patients with hematologic cancers infections.

### 4.1 Human neutrophil lipocalin

Human neutrophil lipocalin (HNL) was first identified and purified in human neutrophils and can also be produced by epithelial cells, HNL is also called neutrophil gelatinase-associated lipocalin (NGAL) or lipocalin 2 (Xu and Venge, 2000; Venge, 2018). The primary source of serum HNL concentrations is activated neutrophils, except in patients with acute kidney injury, where HNL is elevated (Marakala, 2022). Overexpression of free HNL, a prognostic marker for CML, is observed in blood cells from patients with all types of leukemia (Bauvois and Susin, 2018). It has also been found that HNL expression is lower in AML and MDS than in controls (Cho and Cha, 2020). HNL is also differentially expressed in various tumors, with higher levels in adenocarcinomas of the lung, colon, and pancreas, and negative immunostaining for HNL in lymphomas and thymic tumors (Friedl et al., 1999). The likelihood of HNL as an acceptable test to rule out bacterial infection is very high, and the likelihood of acute bacterial infection at concentrations below 155 μg/L is very low (Venge, 2018). HNL has better clinical performance in differentiating between bacterial and viral infections compared to CRP, CD64, and PCT (Venge et al., 2015). HNL has advantages in differentiating between bacterial and viral infections and may reduce the misuse of antibiotics (Ling Lundström et al., 2023). HNL did not differ in G- versus G+ infections, but rose more markedly in bloodstream infections and abdominal infections (Fang et al., 2020). Currently, HNL is more commonly used for early screening for leukemia, lymphoma, and renal insufficiency in patients with MM (Chae et al., 2015; Sherief et al., 2017; Latoch et al., 2020). Given that HNL can be a rapid test and help identify bacterial and viral infections, the use of HNL in hematologic cancer infections can be further investigated.

### 4.2 Serum amyloid A

Serum amyloid A (SAA) is a generic term for a family of proteins with 103-104 amino acids encoded by different genes. SAA is a marker of the acute phase response (APR), a conservative response in vertebrates to surgery, cancers, infection, and tissue injury (Sun and Ye, 2016). SAA is present in the blood of healthy individuals, but levels are usually low (20-50 µg/mL), however, SAA levels can rise as much as 1,000-fold 24 hours after the start of an APR and decline rapidly when the stereotypical APR pattern disappears (Sack, 2018). SAA has been associated with prognosis in certain lymphomas. SAA is a marker of poor prognosis in diffuse large B-cell lymphoma (DLBCL), and in patients with relapsed/refractory DLBCL, high expression of the SAA protein is significantly associated with B-type symptoms, high levels of lactate dehydrogenase (LDH), and non-germinal center B cells (non-GCB) (Liu H. et al., 2022). Patients with aneurysmal subarachnoid hemorrhage (aSAH) who develop infections during hospitalization already have significantly elevated concentrations of SAA on admission, with 90.9 μg/mL as the threshold, and measurement of SAA can be an effective method for detecting patients susceptible to infections during hospitalization after aSAH (Azurmendi et al., 2015). In the study of infection indicators in neonatal sepsis, the investigators collected blood samples at three time points (0 h, 12-24 h, and 48-72 h) and found that IL-6 and SAA were elevated in the confirmed sepsis group (CSG), and the suspected sepsis group (SSG) compared to the control group at 0 h, and SAA was elevated at 12-24 h, IL-8 and SAA were elevated at 48-72 h (Bengnér et al., 2021). SAA has the most favorable kinetic properties in the diagnosis of neonatal sepsis. The researcher divided children with respiratory tract infections into bacterial infection group, non-bacterial infection group, and control group, and found that serum SAA, PCT, and CRP levels in the bacterial infection group were 281.34 ± 42.45, 3.28 ± 1.01, and 42.67 ± 11.02, respectively, and serum SAA, PCT, and CRP levels in the bacterial infection group were higher than those in the non-bacterial infection group and in the healthy children, and the difference was statistically significant (p < 0.05) (Yin and Mo, 2022). The application of SAA in the prediction of infection in hematological cancer patients is somewhat limited because SAA is also associated with APRs such as tumors and surgeries, and the combination of SAA with other biological indicators such as PCT, CRP, and cytokines as a judgment of infection is a good choice.

### 4.3 Heparin-binding protein

Heparin-binding protein (HBP) is a neutrophil-derived granule protein located in the secretory vesicles of neutrophils (Honore et al., 2019). HBP is released early in the response to infection, with neutrophils releasing 89% of total HBP within 30 minutes of phagocytosis of bacteria, a proportion much higher than that of other neutrophil granule proteins (Fisher and Linder, 2017). HBP brings more leukocytes to the site of infection, and it acts as a chemoattractant for macrophages, T lymphocytes, and neutrophils (Fisher and Linder, 2017). Plasma HBP levels differentiate patients with severe sepsis from those with mild infections, and in some patients with severe sepsis, HBP levels are elevated before the onset of infectious shock, and in some patients as early as 12 hours before the onset of hypotension (Fisher and Linder, 2017). The sensitivity and specificity of HBP for the diagnosis of severe sepsis were 87% and 95%, respectively, using 15 ng/mL as the threshold value (Fisher and Linder, 2017). Paulsson M et al. collected bronchoalveolar lavage fluid from 14 patients with pneumonia and 14 controls with a median HBP of 14,690 ng/mL for pneumonia and 16.2 ng/mL for controls, they went on to collect bronchial wash samples from 10 patients with pneumonia and 10 mechanically ventilated controls with a median HBP of 9002 ng/mL for pneumonia and 7.6 ng/mL for the controls (Paulsson et al., 2021). In pediatric patients with community-acquired pneumonia (CAP), substantially elevated serum HBP (≥60 ng/mL) is strongly associated with severe or complicated CAP and poor prognosis (Li et al., 2023). HBP levels were significantly higher in patients with severe sepsis compared to those with non-severe sepsis, and a decrease in HBP levels within 72 hours was significantly associated with a decrease in in-hospital mortality (Liu P. et al., 2023). A meta-analysis found that HBP was diagnostic for sepsis with an overall sensitivity of 0.85, an overall specificity of 0.91, and that HBP levels were significantly elevated at least 24 hours before the diagnosis of sepsis (Wu et al., 2021). Patients with critically ill coronavirus disease 2019 (COVID-19) infection and organ dysfunction had significantly higher HBP compared to those without organ dysfunction (Mellhammar et al., 2021). In neutropenic patients with fever, there are still patients with detectable HBP, patients with detectable HBP have significantly higher neutrophil counts than patients with HBP below the lower limit of detection (LOD), and not all patients with HBP above the LOD have detectable neutrophils (Fisher et al., 2022). The results of this study suggest that the application of HBP to predict infection in patients who develop granulocyte deficiency after chemotherapy for hematologic cancers is also turning out to be possible. However, it should be noted that acute monocytic leukemia cells can release HBP, which may cause false positives (Fisher et al., 2022). The baseline levels of HBP in other types of hematologic cancers and whether HBP can be used as a biomarker after the development of a serious infection deserve to be further investigated.

### 4.4 Ferritin

Ferritin is an intracellular spherical iron storage protein consisting of a combination of 24 subunits of two types, heavy chain ferritin (HFn) and light chain ferritin (LFn) (Song et al., 2021). Ferritin, a molecule that safely retains excess intracellular iron in the core of its shell, was the first iron metabolism-regulating protein to be discovered (Yanatori et al., 2023). In addition to storing iron, new roles for ferritin are being discovered, and dysregulated iron metabolism has been linked to a variety of cancers. Dysregulation of ferritin levels has been reported in many cancers, including breast cancer, glioblastoma and prostate cancer (Alkhateeb et al., 2013; Schonberg et al., 2015; Lu et al., 2020). Ferritin affects various cellular processes such as proliferation and apoptosis in cancer cells (Shesh and Connor, 2023). Ferritin is a positive APR with high concentrations in both intracellular and extracellular mediators, and the role of ferritin in iron homeostasis in the context of inflammation is important for the body’s defense against infection, injury, and cancer (Mahroum et al., 2022). In hematologic disorders, ferritin serves as a biomarker for the occurrence of cytokine release syndrome (CRS) in CAR-T therapy and as an important indicator for the diagnosis of hemophagocytic lymphohistiocytosis (HLH), all of which reflect the role of ferritin as an APR (Griffin et al., 2020; Wei et al., 2023). Acute infections (both bacterial and viral) may result in hyperferritinemia (Kossiva et al., 2012; Xu et al., 2020; Fauter et al., 2022; Zhao et al., 2024). In fact, ferritin may be an important source of iron for bacteria during infection, bacterial virulence and ability to proliferate is related to the availability of free iron in the environment, and in order to establish an infection, bacteria must acquire iron from the host, and iron overload is associated with an increased risk of infection (Griffiths, 1991; Gehrer et al., 2023; Levinson et al., 2023). Ferritin levels can be expected to peak within 3-4 days after an inflammatory stimulus (Karschnia et al., 2019; Levinson et al., 2023). Ferritin is a key marker of macrophage activation, and hyperferritinemia (ferritin ≥500 ng/mL) was seen in 112 of 494 influenza patients (23%) (39/68 or 57% in the poor prognosis group; 73/426 or 17% in the remaining patients, P < 0.0001), and hyperferritinemia was associated with the odds of a poor prognosis in influenza patients associated with a five-fold increase (Lalueza et al., 2020). The overall diagnostic accuracy of ferritin in predicting poor prognosis was 79.3% (Lalueza et al., 2020). COVID-19 can likewise lead to hyperferritinemia (Mahroum et al., 2022). Hyperferritinemia may be a risk factor for IFI in hematologic malignancies. A Greek study enrolled 98 patients (76 with AML and 22 with MDS), 22 of whom developed IFI, and found that the presence of AML was associated with an increased risk of IFI, whereas indicators of iron overload were not an independent risk factor for IFI (Apostolidi et al., 2023). Ferritin, an ARP, is increased in many inflammatory states, and it may serve as a biomarker for CRS, HLH in hematologic disorders, but when used as a predictor of infection, ferritin is affected by a variety of factors including IL-6, IL-10, IL-18, and IFNγ (Karakike and Giamarellos-Bourboulis, 2019). Although the prospects for ferritin’s use in hematologic cancer infections are limited, it still warrants further exploration.

### 4.5 Tumor necrosis factor-related apoptosis-inducing ligand

Tumor necrosis factor-related apoptosis-inducing ligand (TRAIL) is a cytokine of the TNF superfamily, and TRAIL is not only involved in inhibiting tumor growth, but also in neutrophil apoptosis, attenuating inflammatory responses, infection control, and regulation of innate and adaptive immune responses (McGrath et al., 2011; Gyurkovska and Ivanovska, 2016; Marks et al., 2020). Van Houten et al. tested a combination of TRAIL, CRP, and plasma interferon-gamma protein-10 (IP-10) to differentiate between bacterial and viral infections in children, and they found that this assay was more effective than PCT in identifying bacterial and viral cases, with a sensitivity of 86.7% and a specificity of 91.1% (van Houten et al., 2017). IP-10 is a chemokine that has been shown to be involved in the response to bacterial infections (Azzurri et al., 2005). Halabi S et al. integrated the blood levels of three immune proteins, TRAIL, CRP and IP-10, into a single score to differentiate between bacterial and viral infections in adults and found that this method had a sensitivity of 98.1%, a specificity of 88.4% and a negative predictive value of 98.8% for bacterial infections (Halabi et al., 2023). The main application of TRAIL is to differentiate respiratory bacterial infections from viral infections in combination with other indicators, but no studies related to its use in hematologic cancers have been seen.

These indicators have been used in clinical work as biomarkers of infection, but there is a lack of research on their role in predicting infection in hematologic cancers, which is a future direction for research. Some of these indicators or combinations of indicators can distinguish between bacterial and viral infections, which we summarize in **Table 2**.

**Table 2:** Differentiation of bacterial infections from viral infections.

Biomarkers	Changes in biomarkers	Other situations	References
HNL	The likelihood of bacterial infection is low when the HNL is less than 155 μg/L	HNL is elevated in acute kidney injury and some advanced cancers	(Venge, 2018)
SAA, PCT, CRP	Significantly higher SAA, PCT and CRP in patients with bacterial infection compared to patients with non-bacterial infection	SAA rises in some APR cases such as tumors and surgeries	(Yin and Mo, 2022)
HBP	HBP is elevated in bacterial infections. The sensitivity and specificity of HBP for the diagnosis of severe sepsis were 87% and 95%, respectively, using 15 ng/mL as the threshold value	HBP can be increased in viral infections combined with severe organ impairment	(Fisher and Linder, 2017; Mellhammar et al., 2021)
TRAIL, CRP, and IP-10	This combination had a sensitivity of 86.7% and a specificity of 91.1% for differentiating bacterial infections from viral infections.	It increased the diagnostic classification rate of viral and bacterial cases by 6.3% and 5.4%, respectively	(van Houten et al., 2017)

## 5 Conclusion

Infection is one of the major causes of death in patients with hematologic cancers, and many patients with FN suffer from antibiotic overuse; our review summarizes the biomarkers currently available for use in patients with hematologic cancers, which are used to help clinicians make early determinations of the type of infection in their patients, to make antimicrobial medication adjustments, and to improve patient survival. Biomarkers that are already used in blood cancer patients: PCT, cytokines, presepsin, etc. can be used as predictors of bacterial infections, while BDG and GM have an important place in fungal prediction. The importance of mNGS as a biomarker of infection is growing, and with the declining cost of its testing, it could be used in the future as a routine test for hematological cancer patients with FN. We also summarized some biomarkers of infection that have not yet been applied in patients with hematologic cancers with FN, which could serve as future research directions. Of course, the combination of various biomarkers and the integration of the clinical reality of the patient are even more important in the determination of the type of infection.
